# Subjective Proximity to Green Spaces and Blood Pressure in Children and Adolescents: The CASPIAN-V Study

**DOI:** 10.1155/2020/8886241

**Published:** 2020-12-11

**Authors:** Behzad Abbasi, Mohammadali Pourmirzaei, Sanam Hariri, Ramin Heshmat, Mostafa Qorbani, Payam Dadvand, Roya Kelishadi

**Affiliations:** ^1^Faculty of Medicine, Isfahan University of Medical Sciences, Isfahan, Iran; ^2^Department of Pediatrics, Isfahan University of Medical Sciences, Isfahan, Iran; ^3^Chronic Diseases Research Center, Endocrinology and Metabolism Population Sciences Institute, Tehran University of Medical Sciences, Tehran, Iran; ^4^Non-Communicable Diseases Research Center, Alborz University of Medical Sciences, Karaj, Iran; ^5^ISGlobal, Barcelona, Spain; ^6^Universitat Pompeu Fabra (UPF), Barcelona, Spain; ^7^CIBER Epidemiología y Salud Pública (CIBERESP), Madrid, Spain; ^8^Child Growth and Development Research Center, Research Institute for Primordial Prevention of Non-Communicable Disease, Isfahan University of Medical Sciences, Isfahan, Iran

## Abstract

Evidence favoring a beneficial association between greenness and blood pressure (BP) in adults is accumulating. However, children and adolescents have been understudied accordingly. Methodologically, the data on “exposure” to residential green spaces are commonly satellite-derived, including rare existing studies on the relationship between proximity to green spaces and BP in children. Despite perfectly obliterating subjective biases, remote sensing methods of greenness data collection fail to address pragmatic interaction with such settings. This study aimed to assess the relationship between subjective proximity to green spaces and average/elevated BP in children. Through our study, systolic and diastolic BPs of 12,340 schoolchildren living in CASPIAN-V study areas were examined and recorded. We performed surveys to obtain the data on their proximity to green spaces, defined as having access to such spaces within a 15-minute walk from their homes. Linear mixed-effects models with BP as the outcome variable and the measure of exposure to green spaces as fixed-effect predictor were applied. The analysis was adjusted for several covariates. We found that perceived residential proximity to green spaces was associated with −0.08 mmHg (95% confidence intervals (CIs): −0.58, 0.41; *p* value = 0.72) reduction in systolic BP and −0.09 (95% CIs: −0.49, 0.31; *p* value = 0.66) reduction in diastolic BP. We also observed statistically nonsignificant odds ratio of 1.03 (95% CIs: 0.76, 1.39), 0.96 (95% CIs: 0.80, 1.16), and 0.98 (95% CIs: 0.82, 1.16) for isolated systolic/diastolic hypertension and hypertension, respectively. Our observations remained consistent after adjustment for height, parental employment, low birth weight, parental obesity, single parent, and breastfeeding. In conclusion, subjective proximity to green spaces might not be associated with a lower mean BP in children. Well-designed studies applying both subjective and objective data should be performed to elaborate on the relationship further.

## 1. Introduction

Over the past decades, the prevalence of noncommunicable diseases (NCDs) has risen strikingly, indicating an alarming worldwide epidemic [1]. Consequently, NCDs and primarily cardiovascular diseases have taken over as the chief cause of morbidity and mortality, accounting for 71% of annual deaths globally [1]. Accumulating evidence is shedding light on the tracks of NCDs and specifically cardiovascular diseases in the preadulthood, highlighting the significance of early environmental exposures [2–8]. Accordingly, childhood and adolescence could be considered opportunities to prevent NCDs from happening later in life through environmental and behavioral interventions.

As evidenced, habitual factors (i.e., diet, physical activity, use of tobacco, and alcohol consumption), hereditary susceptibility, and stress, as well as sociodemographic/economic status and cultural alterations, contribute to the conditions predisposing a person to cardiovascular disease, embodying elevated blood glucose and lipids, obesity, and most notably high blood pressure (BP) [9].

In addition to classically established risk factors for high BP and hypertension (HTN), the role of urban environmental exposures, namely, disposal to air and noise pollutions, has gained attention owing to the recent research studies [10–15]. Besides, exposure to green environments may diminish the risk of high BP and HTN in adults, presumably through enhancing physical activity and alleviating stress as well as being subject to air/noise pollutants [16–19].

The benefits of green spaces in children and adolescent are relatively well-documented; exposure to greenness has been associated with mental and behavioral advantages [20–22], less asthma prevalence and symptoms [23, 24], better lung functionality [25], and reduction in insulin resistance [26] and obesity [27, 28]. Howbeit, the evidence linking the green space exposure to BP in children and adolescents has faced scarcity. Studies have indicated that schoolyard “greening” and green exercise are associated with lower systolic (SBP) and diastolic blood pressure (DBP) in children [29, 30]. In a recent attempt, Xiao et al. have demonstrated higher greenness around school associates with lower SBP and lower odds of HTN [31]. Data on the residential greenness and BP in children, however, are confined to the sole existing study concluding that children residing in urbanized areas tend to be prone to higher mean systolic and diastolic BP levels than those inhabiting green neighborhoods [32].

While previous studies evaluated the proximity to green spaces mostly by analyzing the data obtained from satellites, reported as normalized difference vegetation index (abbr. NDVI) in a considerable number of cases, the relationship between perceived proximity to green spaces and children wellbeing yet has not been described in the medical literature. Nevertheless, the perception of the adjacent residential green spaces might link the existence of such spaces to experiential contact with them.

Conducting this study, we aim to evaluate the association between subjective proximity to green spaces and BP in children and adolescents. Towards this purpose, we also measure these associations variation by socioeconomic status, level of urbanity, and sex.

## 2. Materials and Methods

### 2.1. Study Area

Iran is a country located in west Asia spanning 1,648,195 km^2^ with a total population of 79.9 million (density: 48.49 persons/km^2^), *f* which 59.1 million (74%) live in urbanized areas [33]. Approximately 85% of Iran's lands is rather arid or semiarid [34] consisting of 14% arable land, 8% forest, 55% natural pastures, and 23% desert [35, 36].

### 2.2. Study Setting

This study was conducted as a part of the fifth survey of a national surveillance program entitled the Childhood and Adolescence Surveillance and PreventIon of Adult Non-communicable disease study (CASPIAN-IV, 2015). Applying a multistage/stratified cluster sampling method, we surveyed a population-based sample of 14,400 schoolchildren aged 7–18 in urban and rural areas of thirty provinces of Iran. A detailed description of the sampling and data collection methods is published elsewhere [37]. In brief, from each province, 480 students were recruited. Sampling within each province was carried out according to the student's residence (urban or rural) and education level (primary or secondary school) using the proportional to size method with an equal sex ratio.

Consequently, the ratio of participants in urban and rural areas and each grade in each province was proportional to the number of schoolchildren studying in urban and rural areas and in each grade in that province. A cluster sampling method was then used to achieve the required sample size for rural and urban areas and each province's grade. Clusters were determined at school levels with ten schoolchildren (and their parents) in each cluster, resulting in 48 clusters in each province.

This study was approved by The Research and Ethics Council of Isfahan University of Medical (Project Number: 194049). Written informed consent and verbal consent were obtained from the parents and schoolchildren, respectively, after thorough clarification of the study aims and protocols.

### 2.3. Questionnaire Data

Two discrete questionnaires were designated for schoolchildren and their parents. Data on subjective proximity to green spaces were acquired through the schoolchildren's questionnaire, while data on covariates and mediators were obtained from parents. In-person interviews were conducted for each of the research participants to obtain these data. The details of questionnaires used in CASPIAN-V and circumstances under which the interviews were conducted have been described elsewhere [37].

#### 2.3.1. Subjective Proximity to Green Spaces

In the section of the green space in the questionnaire, schoolchildren were questioned if they have access to a green space following a 15-minute walk from their home with possible answers as “yes,” “no,” and “I do not know.” Green spaces were defined as parks, land covered with growing trees, agricultural fields, gardens, etc.

#### 2.3.2. Covariate and Mediator Data

Data on sociodemographic characteristics (e.g., maternal and paternal educational attainment, employment, marital status, and homeownership), family medical history (e.g., history of HTN in students first and second relatives), and parental anthropometric measures (e.g., weight and height) were obtained by questionnaires.

#### 2.3.3. Blood Pressure

We applied the auscultatory method to measure the children's BP: schoolchildren's BP was evaluated on the right arm supported to the heart level while sitting. A standard mercury sphygmomanometer with a convenient cuff size was utilized for each, and a stethoscope was placed over the brachial artery to monitor Korotkoff sounds. The blood pressure was measured twice following a 5-minute interval, and the average was recorded [38].

We defined systolic HTN and diastolic HTN as average systolic BP (SBP) and diastolic BP (DBP) that is ≥95^th^ percentile for sex, age, and height [38].

HTN was defined as the existence of one/both of the mentioned conditions. Prehypertension was described as mean SBP/DBP levels that are ≥90^th^ percentile but less than 95^th^ percentile [38].

However, in line with Kaelber and Pickett's simplified definition of elevated BP in children, we used their cutpoints corresponding to the lower limit of height (5th percentile) in the prehypertensive BP range (≥90^th^ percentile) for a given age and gender [39, 40].

### 2.4. Main Analyses

Because of the data's multilevel nature, we used linear mixed-effects models with BP as the outcome variable, each level of green space exposure (one-at-a-time) as the fixed-effect predictor, and recruitment center and cluster as the random effects. The analyses were adjusted for several covariates identified *a priori:* child's age, sex, parental and family history of HTN (yes/no), exposure to environmental tobacco smoke (yes/no), urbanity (urban/rural), and indicators of socioeconomic status (SES) including parental educational attainment (highest degree attained by either parent: primary school/secondary school/university), house ownership (owning the house: yes/no), and school type (private/public).

### 2.5. Sensitivity Analyses

We used systolic and diastolic pressures divided by height as alternative outcomes [39, 40]. We also further adjusted our analyses for parental employment (unemployed/employee/self-employed), low birth weight (yes/no), parental obesity (at least one parent being obese: yes/no), single parent (yes/no), and breastfeeding (at least six of exclusive breastfeeding: yes/no). For the main analyses, we excluded those participants answering “I do not know” to our question (*N* = 289). We conducted a sensitivity analysis by including these participants as another group in our proximity variable, resulting in three categories: “yes,” “no,” and “I do not know.” To evaluate the influence of age on our findings, especially in terms of the accuracy of the reported use and proximity of green space in younger children, we stratified our analyses based on our participants median age (i.e., 12 years old).

### 2.6. Further Analyses

We developed logistic mixed-effects models using systolic HTN, diastolic HTN, and HTN (previously defined in Section 2.3) as an outcome variable with an equal set of random/fixed-effect exposure and covariates main analyses.

## 3. Results

In the present study, 12,340 students out of 14,440 and one of their parents completed the survey. Our participants consisted of 6,092 (49.37%) girls and 6,248 (50.63%) boys. The median and interquartile range for age were 12 and 5, respectively. 8,807 (71.37%) were from urban areas, while 3,533 (28.63%) had rural backgrounds. 91.60% of the participants attended public school. In our analysis, we found that 8,035 (65.11%) of our participants had proximity to green spaces, while 4,305 (34.89%) did not (Table 1). Data on homeownership/parental educational status and familial history of HTN could be found in Tables 2 and 3, respectively.

Perceived residential proximity to green spaces was associated with −0.08 mmHg (95% confidence intervals (CIs): −0.58, 0.41, *p* value = 0.72) reduction in SBP and −0.09 (95% CIs: −0.49, 0.31, *p* value = 0.66) reduction in DBP; however, none of the associations was statistically significant.

### 3.1. Further Analyses

Perceived residential proximity to green spaces did not have any statistically significant association with measures of HTN as we observed nonsignificant odds ratio (95% CIs) of 1.03 (0.76, 1.39), 0.96 (0.80, 1.16), and 0.98 (0.82, 1.16) for systolic HTN, diastolic HTN, and HTN, respectively.

### 3.2. Sensitivity Analyses

Using SBP and DBP divided by height did not change our findings significantly. Similarly, our observations remained consistent after further adjustment of parental employment analyses, low birth weight, parental obesity, single parent, and breastfeeding. Likewise, applying the alternative classification of perceived proximity to green spaces did not considerably change our results.

## 4. Discussion

To our knowledge, this study is the first to report on the association between perceived proximity to green spaces and BP in children and adolescents. Recruiting questionnaires to assess perceived proximity to green spaces and physical examinations to measure BP, we conducted this study based on a large nationally representative sample of Iranian schoolchildren [37]. Although we observed a reduction in mean SBP and DBP in association with subjective proximity to green spaces, none attained statistical significance. Similarly, we observed no statistical significance regarding the association between such exposure and elevated BP.

Evidence on the relationship between exposure to greenness and BP is emerging, and documentation supporting the association between green space exposure and BP in children and adolescents is phenomenal. A study carried out on children living in the Munich and Wesel study areas of the German GINIplus and LISAplus birth cohorts confirmed that children residing in the urbanized areas tend to have higher mean SBP and DBP levels compared to those living in green neighborhoods [32]: SBP of children living at low and moderate green residences was 0.90 ± 0.50 mmHg and 1.23 ± 0.50 mmHg higher, compared to the SBP of children living in areas of high greenness (*p*=0.073 and  *p*=0.041, respectively). Similarly, analyzing DBP resulted in resembling favorable results [32]. In the most recent attempt, Xiao et al. probed the greenness around seven schools in China and its association with BP in children [31]. Through the course of their study, they observed that a 0.1 unit elevation in green space exposure was significantly associated with a −1.39 (95% CI: −1.86, −0.93) mmHg reduction in SBP lower odds of HTN (OR = 0.76, 95% CI: 0.69, 0.82); the associations were more robust in children with a higher BMI. However, in their model, no significant association was observed between greenness and DBP. The results of our study, however, were inconsistent with the mentioned studies as we observed no statistically significant association between subjective proximity to green spaces and neither SBP nor DBP.

Nevertheless, our work was in line with Bloemsma et al. study, unsuccessful in characterizing an association between exposure to green space and cardiometabolic health in adolescents [41]. Likewise, Gutiérrez-Zornoza et al. concluded that residential distance to green spaces does not determine the cardiometabolic risk in schoolchildren aged 10 to 12 [42]. Although the mentioned studies did not focus on BP as the primary endpoint, they recruited it as a cardiometabolic risk criterion.

Additionally, a growing number of studies have investigated the longitudinal relationship between early green space exposure and BP status in adulthood. For instance, through work on New England Family Study data, Jimenez and colleges found that residing a mile away from a green space at birth was associated with higher SBP (5.6 mmHg; 95% CI: 0.7, 10.5) and DBP (3.5 mmHg; 95% CI: 0.3, 6.8) in adulthood. Moreover, one extra residential green space at birth was also associated with lower adulthood DBP (−0.2 mmHg, 95% CI: −0.4, −0.02) [43]. In another longitudinal study on 178 twins, Bijnens and colleges noted an association between interquartile elevation in exposure to residential greenness (1000 m radius) with lower adult SBP at night (3.59 mmHg; 95% CI: −6.0, −1.23). Shifting “early exposure” to the next level, a recent study has figured that an interquartile increase in the prenatal residential greenness was significantly associated with 1.2 mm Hg reduction in neonatal both SBP and DBP (95% CI: −2.5, 0.1; and 95% CI: −2.4, −0.0, respectively).

Different studies have applied various metrics to evaluate the level of exposure to green spaces, most of which depend on actual satellite-derived data. On the contrary, measuring the use of such spaces has drawn attention recently, which majorly stems from the fact that the existence of such spaces does not necessarily imply the use of them. Simultaneously, the accumulating evidence has acknowledged the mediating role of interaction with green spaces in their trajectory of health beneficence.

Cognition toward residential environmental may provoke actual interaction with them [44, 45].

We hypothesized that the perception of green spaces is the link between the existence and use of such spaces. In line with us, it has been indicated that subjective proximity to green spaces predicts green space visits in adolescents more precisely compared with the objective extent of residential green space [46]. To the best of our knowledge, not a single study yet has addressed the perceived proximity to green spaces in association with BP, neither in adults nor children/adolescents. However, in our model, we could not represent a significant logical association between the perception of residential green spaces and blood pressure in children or adolescents in Iran.

**Figure 1:**
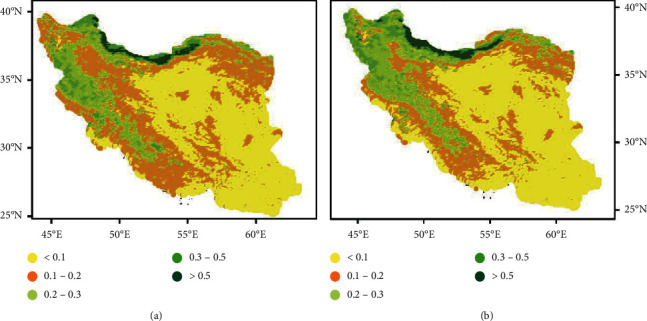
Mean NDVI in Iran, 1988–2015 (used with permission from Fakharizadehshirazi et al. [49]): (a) spring; (b) summer.

**Figure 2:**
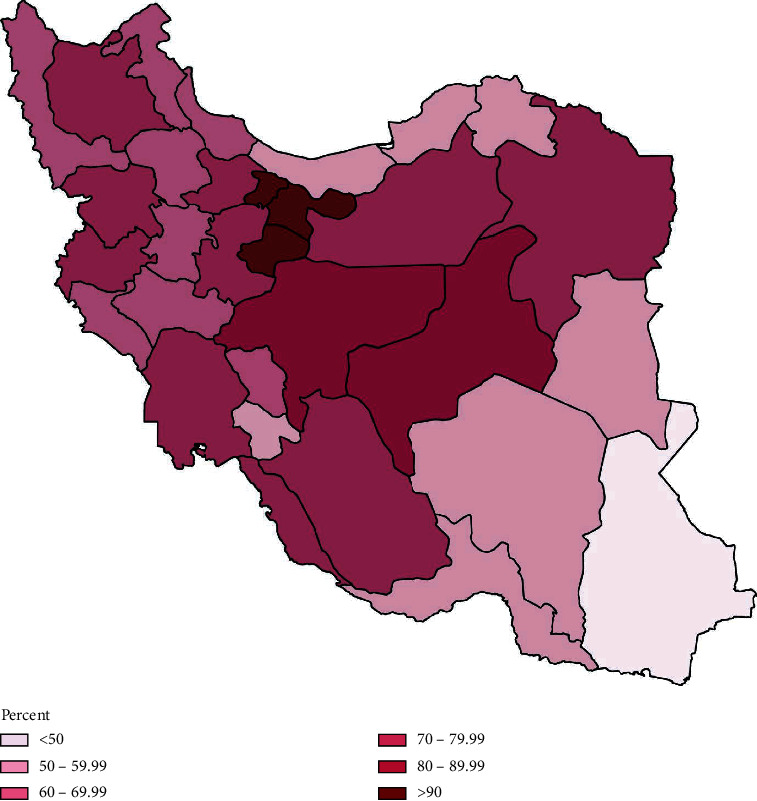
Map of urbanity in Iran by provincial divisions. Data were extracted from the statistical center of Iran [33].

The distribution of green spaces in Iran (Figure 1) encounters two major obstacles resulting in inequality. First, a historical trend of extreme climatic dispersion—varying from hot arid to subtropical—with average annual precipitation ranging from zero in the central desert of Iran to over 1,250 mm in sub-Caspian coasts contributing to extreme natural greenness diversity with an in-land area consisting of 14% arable land, 8% forest, 55% natural pastures, and 23% desert [35, 36]. And second, a significant disproportion in terms of urbanization level observed between provinces throughout Iran, which in turn, deprive populations of regular interaction with green settings (Figure 2) [47]. As noted, by 2016, 74% of Iran's population lived in urban regions, moderately above the worldwide average (58%) [48], which may contribute to the green space deprivation compared to the international trend.

Previous studies, applying an experimental design, mainly focused on exposure to green spaces in both laboratory settings and nature in adults, aiming to assess the impact of confrontation on physiological stress parameters (for a review, see [50]). The mechanisms involved in stress consolation due to green space exposure have been outlined, particularly by “Ulrich's psycho-evolutionary theory” [51]. Based on the fore-said theory, humans' high adaptability to natural environments versus urban areas emerges from his extended evolution in the mentioned settings. Accordingly, being exposed to green spaces develops multiple-system responses ending in reduced sympathetic function measures (e.g., lower Cortisol level, lower BP, and lower heart rate) [51].

Following Ulrich's theory, some studies approved the link between subjection to images of greenness in laboratory setting and walking through them and a rare decrease in BP in adults; such studies mostly gave credit to the theory above in a mechanistic point of view [52–54].

## 5. Limitations

Our cross-sectional analysis by design had a limited capability to establish a causal relation. We did not have data on the geocoded residential address of the study participant. It was therefore not possible to assess objective residential proximity to green spaces, which could enable us to compare the effects of subjective and objective proximity to green spaces on blood pressure. We also did not have data on neighborhood socioeconomic status, which could have influenced our findings. However, our analyses for a wide range of household socioeconomic status indicators could have partially addressed this.

## 6. Conclusions

We found that subjective proximity to green spaces had no perceptible association with a lower mean BP in children and adolescents through the course of the study. Likewise, no significant link existed between such proximity and SBP, DBP, and HTN in the mentioned target group. The associations remained insignificant after adjusting the model for parental employment, birth weight, parental obesity, single parent, and breastfeeding status. We recommend further thorough investigations applying subjective and objective means of data collection, enabling the comparison between the association between different metrics of exposure to green spaces.

## Figures and Tables

**Table 1:** Sociodemographic characteristics of study participants (students) and green space subjection status.

Variable	Description
Sex	
Female	6,092 (49.37%)
Male	6,248 (50.63%)
Living area	
Urban	8,807 (71.37%)
Rural	3,533 (28.63%)
School type	
Public	11,303 (91.60%)
Private	940 (7.62%)
Missing	97 (0.79%)
Subjective green space proximity	
No	4,305 (34.89%)
Yes	8,035 (65.11%)
Passive smoker	
No	7,094 (57.49%)
Yes	5,246 (42.51%)

**Table 2:** Education and homeownership of the participants (parents).

Variable	Description
Parental education	
No or primary school	3,418 (27.70%)
Secondary school	6,594 (53.44%)
University	2,176 (17.63%)
Missing	152 (1.23%)
Homeownership	
No	2,084 (16.89%)
Yes	10,146 (82.22%)
Missing	110 (0.89%)

**Table 3:** History of HTN in parents, grandparents, and second-degree relatives (i.e., aunts and uncles) of students.

Father	
No	10,972 (88.91%)
Yes	1,093 (8.86%)
Missing	275 (2.23%)
Mother	
No	10,807 (87.58%)
Yes	1,325 (10.74%)
Missing	208 (1.69%)
Grandparents	
No	5,999 (48.61%)
Yes	5,969 (48.37%)
Missing	372 (3.01%)
Aunts/uncles	
No	9,594 (77.75%)
Yes	2,507 (20.32%)
Missing	239 (1.94%)

## Data Availability

As this study was conducted as a part of a national surveillance program (CASPIAN-V), the data are not freely accessible.

## References

[1] Kyu H. H., Abate D., Abate K. H. (2018). Global, regional, and national disability-adjusted life-years (DALYs) for 359 diseases and injuries and healthy life expectancy (HALE) for 195 countries and territories, 1990–2017: a systematic analysis for the global burden of disease study 2017. *The Lancet*.

[2] Balbus J. M., Barouki R., Birnbaum L. S. (2013). Early-life prevention of non-communicable diseases. *The Lancet*.

[3] Hanson M. A., Gluckman P. D. (2015). Developmental origins of health and disease—global public health implications. *Best Practice and Research: Clinical Obstetrics & Gynaecology*.

[4] Yang L., Sun J., Zhao M., Liang Y., Bovet P., Xi B. (2020). Elevated blood pressure in childhood and hypertension risk in adulthood: a systematic review and meta-analysis. *Journal of Hypertension*.

[5] Hao G., Wang X., Treiber F. A., Harshfield G., Kapuku G., Su S. (2017). Blood pressure trajectories from childhood to young adulthood associated with cardiovascular risk: results from the 23-year longitudinal Georgia stress and heart study. *Hypertension*.

[6] Allen N. B., Siddique J., Wilkins J. T. (2014). Blood pressure trajectories in early adulthood and subclinical atherosclerosis in middle age. *JAMA—Journal of the American Medical Association*.

[7] Rosner B., Cook N. R., Daniels S., Falkner B. (2013). Childhood blood pressure trends and risk factors for high blood pressure: the NHANES experience 1988–2008. *Hypertension*.

[8] Lee J. W., Kim N., Park B., Park H., Kim H. S. (2020). Blood pressure trajectory modeling in childhood: birth-cohort study. *Clinical Hypertension*.

[9] World Health Organization (2017). *Cardiovascular Diseases (CVDs)*.

[10] Li N., Chen G., Liu F. (2020). Associations between long-term exposure to air pollution and blood pressure and effect modifications by behavioral factors. *Environmental Research*.

[11] Hou J., Duan Y., Liu X. (2020). Associations of long-term exposure to air pollutants, physical activity and platelet traits of cardiovascular risk in a rural Chinese population. *Science of the Total Environment*.

[12] Amini H., Dehlendorff C., Lim Y. H. (2020). Long-term exposure to air pollution and stroke incidence: a Danish nurse cohort study. *Environment International*.

[13] van Kempen E., Casas M., Pershagen G., Foraster M. (2018). WHO environmental noise guidelines for the European region: a systematic review on environmental noise and cardiovascular and metabolic effects: a summary. *International Journal of Environmental Research and Public Health*.

[14] Yang B. Y., Qian Z., Howard S. W. (2018). Global association between ambient air pollution and blood pressure: a systematic review and meta-analysis. *Environmental Pollution*.

[15] Dzhambov A. M., Dimitrova D. D. (2018). Residential road traffic noise as a risk factor for hypertension in adults: systematic review and meta-analysis of analytic studies published in the period 2011–2017. *Environmental Pollution*.

[16] Brown S. C., Lombard J., Wang K. (2016). Neighborhood greenness and chronic health conditions in medicare beneficiaries. *American Journal of Preventive Medicine*.

[17] Lane K. J., Stokes E. C., Seto K. C., Thanikachalam S., Thanikachalam M., Bell M. L. (2017). Associations between greenness, impervious surface area, and nighttime lights on biomarkers of vascular aging in Chennai, India. *Environmental Health Perspectives*.

[18] Dzhambov A. M., Markevych I., Lercher P. (2018). Greenspace seems protective of both high and low blood pressure among residents of an Alpine valley. *Environment International*.

[19] Jiang J., Chen G., Li B. (2020). Does long-term green space exposure improve hypertension and blood pressure? The Henan rural cohort study. *Lancet Public Health*.

[20] Dadvand P., Hariri S., Abbasi B. (2019). Use of green spaces, self-satisfaction and social contacts in adolescents: a population-based CASPIAN-V study. *Environment International*.

[21] Zhang Y., Mavoa S., Zhao J., Raphael D., Smith M. (2020). The association between green space and adolescents mental wellbeing: a systematic review. *International Journal of Environmental Research and Public Health*.

[22] Amoly E., Dadvand P., Forns J. (2015). Green and blue spaces and behavioral development in barcelona schoolchildren: the BREATHE project. *Environmental Health Perspectives*.

[23] Lovasi G. S., Quinn J. W., Neckerman K. M., Perzanowski M. S., Rundle A. (2008). Children living in areas with more street trees have lower prevalence of asthma. *Journal of Epidemiology and Community Health*.

[24] Li L., Hart J. E., Coull B. A., Cao S. J., Spengler J. D., Adamkiewicz G. (2019). Effect of residential greenness and nearby parks on respiratory and allergic diseases among middle school adolescents in a Chinese city. *International Journal of Environmental Research and Public Health*.

[25] Paciência I., Rufo J. C., Silva D. (2019). School environment associates with lung function and autonomic nervous system activity in children: a cross-sectional study. *Scientific Reports*.

[26] Thiering E., Markevych I., Brüske I. (2016). Associations of residential long-term air pollution exposures and satellite-derived greenness with insulin resistance in German adolescents. *Environmental Health Perspectives*.

[27] Bao W. W., Yang B. Y., Zou Z. Y. (2021). Greenness surrounding schools and adiposity in children and adolescents: findings from a national population-based study in China. *Environmental Research*.

[28] Sanders T. G. (2018). Investigating associations between neighbourhood green space and weight status: a longitudinal study of Australian children aged 4 to 13 years old.

[29] Kelz C., Evans G. W., Röderer K. (2015). The restorative effects of redesigning the schoolyard: a multi-methodological, quasi-experimental study in rural Austrian middle schools. *Environment and Behavior*.

[30] Duncan M. J., Clarke N. D., Birch S. L. (2014). The effect of green exercise on blood pressure, heart rate and mood state in primary school children. *International Journal of Environmental Research and Public Health*.

[31] Xiao X., Yang B. Y., Hu L. W. (2020). Greenness around schools associated with lower risk of hypertension among children: findings from the seven northeastern cities study in China. *Environmental Pollution*.

[32] Markevych I., Thiering E., Fuertes E. (2014). A cross-sectional analysis of the effects of residential greenness on blood pressure in 10-year old children: results from the GINIplus and LISAplus studies. *BMC Public Health*.

[33] Presidency of the I.R.I Plan and Budget Organization (2016). *Selected Findings of the 2016 National Population and Housing Census*.

[34] Squires R. V., Shang Z., Ariapour A. (2017). *Rangelands along the Silk Road: Transformative Adaptation under Climate and Global Change*.

[35] FAO (2016). *FAO in the Islamic Republic of Iran*.

[36] (2016). *World Health Organization, Climate and Health Country Profiles (Iran)*.

[37] Motlagh M. E., Ziaodini H., Qorbani M. (2017). Methodology and early findings of the fifth survey of childhood and adolescence surveillance and prevention of adult non-communicable disease: the CASPIAN-V study. *International Journal of Preventive Medicine*.

[38] Falkner B., Daniels S. R., Flynn J. T. (2004). The fourth report on the diagnosis, evaluation, and treatment of high blood pressure in children and adolescents. *Pediatrics*.

[39] Ma C., Kelishadi R., Hong Y. M. (2016). Performance of eleven simplified methods for the identification of elevated blood pressure in children and adolescents. *Hypertension*.

[40] Kelishadi R., Heshmat R., Ardalan G. (2014). First report on simplified diagnostic criteria for pre-hypertension and hypertension in a national sample of adolescents from the Middle East and North Africa: the CASPIAN-III study. *Jornal de Pediatria (Rio J)*.

[41] Bloemsma L. D., Gehring U., Klompmaker J. O. (2019). Green space, air pollution, traffic noise and cardiometabolic health in adolescents: the PIAMA birth cohort. *Environment International*.

[42] Gutiérrez-Zornoza M., Sánchez-López M., García-Hermoso A., González-García A., Chillón P., Martínez-Vizcaíno V. (2015). Active commuting to school, weight status, and cardiometabolic risk in children from rural areas: the Cuenca study. *Health Education and Behavior*.

[43] Jimenez M. P., Wellenius G. A., James P. (2020). Associations of types of green space across the life-course with blood pressure and body mass index. *Environmental Research*.

[44] Ajzen I. (2011). Behavioral interventions: design and evaluation guided by the theory of planned behavior. https://www.guilford.com/excerpts/mark.pdf.

[45] Ajzen I. (1991). The theory of planned behavior. *Organizational Behavior and Human Decision Processes*.

[46] Bloemsma L. D., Gehring U., Klompmaker J. O. (2018). Green space visits among adolescents: frequency and predictors in the PIAMA birth cohort study. *Environmental Health Perspectives*.

[47] Enayatrad M., Yavari P., Etemad K., Khodakarim S., Mahdavi S. (2019). Determining the levels of urbanization in Iran using hierarchical clustering. *Iranian Journal of Public Health*.

[48] UN/DESA (2018). *World Urbanization Prospects: The 2018 Revision*.

[49] Fakharizadehshirazi E., Sabziparvar A. A., Sodoudi S. (2019). Long-term spatiotemporal variations in satellite-based soil moisture and vegetation indices over Iran. *Environmental Earth Sciences*.

[50] Fong K. C., Hart J. E., James P. (2018). A review of epidemiologic studies on greenness and health: updated literature through 2017. *Current environmental health reports*.

[51] Ulrich R. S., Simons R. F., Losito B. D., Fiorito E., Miles M. A., Zelson M. (1991). Stress recovery during exposure to natural and urban environments. *Journal of Environmental Psychology*.

[52] Brown D. K., Barton J. L., Gladwell V. F. (2013). Viewing nature scenes positively affects recovery of autonomic function following acute-mental stress. *Environmental Science and Technology*.

[53] Ideno Y., Hayashi K., Abe Y. (2017). Blood pressure-lowering effect of Shinrin-yoku (forest bathing): a systematic review and meta-analysis. *BMC Complementary and Alternative Medicine*.

[54] Hartig T., Evans G. W., Jamner L. D., Davis D. S., Gärling T. (2003). Tracking restoration in natural and urban field settings. *Journal of Environmental Psychology*.

